# *Eugenia uniflora* L. Essential Oil as a Potential Anti-*Leishmania* Agent: Effects on *Leishmania amazonensis* and Possible Mechanisms of Action

**DOI:** 10.1155/2013/279726

**Published:** 2013-02-20

**Authors:** Klinger Antonio da Franca Rodrigues, Layane Valéria Amorim, Jamylla Mirck Guerra de Oliveira, Clarice Noleto Dias, Denise Fernandes Coutinho Moraes, Eloisa Helena de Aguiar Andrade, Jose Guilherme Soares Maia, Sabrina Maria Portela Carneiro, Fernando Aécio de Amorim Carvalho

**Affiliations:** ^1^Medicinal Plants Research Center, Federal University of Piauí, 64049-550 Teresina, PI, Brazil; ^2^Laboratory of Pharmacognosy II, Department of Pharmacy, Federal University of Maranhão, 65085-580 São Luís, MA, Brazil; ^3^Postgraduate Program in Chemistry, Federal University of Pará, 66075-900 Belém, PA, Brazil

## Abstract

*Eugenia uniflora* L. is a member of the Myrtaceae family and is commonly known as Brazilian cherry tree. In this study, we evaluated the chemical composition of *Eugenia uniflora* L. essential oil (EuEO) by using gas chromatography-mass spectrometry (GC-MS) and assessed its anti-*Leishmania* activity. We also explored the potential mechanisms of action and cytotoxicity of EuEO. Thirty-two compounds were identified, which constituted 92.65% of the total oil composition. The most abundant components were sesquiterpenes (91.92%), with curzerene (47.3%), **γ**-elemene (14.25%), and *trans*-**β**-elemenone (10.4%) being the major constituents. The bioactivity shown by EuEO against promastigotes (IC_50_, 3.04 **μ**g*·*mL^−1^) and amastigotes (IC_50_, 1.92 **μ**g*·*mL^−1^) suggested significant anti-*Leishmania* activity. In the cytotoxicity determination, EuEO was 20 times more toxic to amastigotes than to macrophages. Hemolytic activity was 63.22% at the highest concentration tested (400 **μ**g*·*mL^−1^); however, there appeared to be no toxicity at 50 **μ**g*·*mL^−1^. While the data show that EuEO activity is not mediated by nitric oxide production, they do suggest that macrophage activation may be involved in EuEO anti-*Leishmania* activity, as evidenced by increases in both the phagocytic capacity and the lysosomal activity. More studies are needed to determine *in vivo* activity as well as additional mechanisms of the anti-*Leishmania* activity.

## 1. Introduction

Protozoan parasites of the genus *Leishmania* are responsible for a spectrum of diseases collectively known as leishmaniasis, which affects the skin, mucous membranes, and internal organs. Over 12 million cases of leishmaniasis have been reported worldwide, with 1 to 2 million new cases being reported annually [1, 2]. Increase in the incidence of leishmaniasis is associated with urban development, deforestation, environmental changes, and increased migration to areas where the disease is endemic [3]. Despite its epidemiological importance, treatment is still performed with chemotherapeutic drugs that are delivered parenterally, require medical supervision, and have many side effects [4, 5].

The need to identify new anti-*Leishmania* compounds that are more effective and less toxic than conventional drugs has motivated research of substances derived from plant species. In this context, essential oils containing a group of secondary metabolites consisting mainly of monoterpenes, sesquiterpenes, and phenylpropanoids have demonstrated proven anti-*Leishmania* activity *in vivo* and *in vitro* on promastigote and/or amastigote forms of *Leishmania*. Included among these are *Croton cajucara* [6], *Ocimum gratissimum* [7], *Copaifera cearensis* [8], *Chenopodium ambrosioides* [9], *Cymbopogon citratus* [10, 11], and *Lippia sidoides* [12]. These studies have shown that essential oils can be a promising source of new drugs with anti-*Leishmania* activity. 

*Eugenia uniflora* L., commonly known as “pitangueira” or Brazilian cherry tree, is a species belonging to the family Myrtaceae, which is native to South America and common in regions with tropical and subtropical climate [13]. In Brazil, it is used in the treatment of digestive disorders [14] and is thought to have anti-inflammatory and antirheumatic activities [15]. It is an aromatic species and its essential oil has pharmacological properties that are well characterized in the literature as antioxidant and antimicrobial [16]. *E. uniflora* has known antihypertensive [17], antitumor [18], and antinociceptive properties [19], and it shows good performance against microorganisms, demonstrating antiviral, antifungal [20], anti-*Trichomonas gallinae* [21], and anti-*Trypanosoma cruzi* properties [22].

Considering the potential pharmacological benefits of *E. uniflora* and the increasing interest in the discovery of essential oils with anti-*Leishmania* activity, the aim of this study was to investigate the anti-*Leishmania* activity of leaf essential oil from this species, the chemical composition of the oil, its cytotoxicity, and possible mechanisms of action.

## 2. Materials and Methods

### 2.1. Chemicals

Dimethyl sulfoxide (DMSO: 99%), anhydrous sodium sulfate, glacial acetic acid, ethanol, formaldehyde, sodium chloride, calcium acetate, zymosan, and neutral red were purchased from Merck Chemical Company (Germany). The *n*-alkane (C_8_–C_20_) homologous series, Schneider's medium, RPMI 1640 medium, fetal bovine serum (FBS), MTT (3-(4,5-dimethylthiazol-2-yl)2,5-diphenyltetrazolium bromide), Griess reagent (1% sulfanilamide in H_3_PO_4_ 10% (v/v) in Milli-Q water), and the antibiotics penicillin and streptomycin were purchased from Sigma Chemical (St. Louis, MO, USA). The antibiotic amphotericin B (90%) was purchased from Cristália (São Paulo, SP, Brazil).

### 2.2. Plant Material

*E. uniflora* leaves were collected (January 2010) from a mature tree in the flowering stage in São Luís (2°30′45.3′′S and 44°18′1.1′′W) in the state of Maranhão in northeast Brazil. The samples were collected from a cultivated plant and its fruits have red color. A voucher specimen (no. 0998/SLS017213) was deposited at the Herbarium “Ático Seabra” of the Federal University of Maranhão, and plant identification was confirmed by botanists of the Herbarium “Murça Pires” from Museu Paraense Emílio Goeldi, Belém, PA, Brazil.

### 2.3. Extraction of the Essential Oil

The plant material was air-dried for 7 days, cut into small pieces, and subjected to hydrodistillation using a Clevenger-type apparatus (300 g, 3 h) to obtain a sesquiterpene-rich essential oil. Once collected, the essential oil was dried over anhydrous sodium sulfate, filtered, and weighed, and then the oil yield was calculated in terms of % (w/w). Its percentage content was estimated based on the plant dry weight by calculating the water content by using a moisture analyzer prior to distillation. EuEO was then stored in a dark flask and refrigerated (at +5°C) until use.

### 2.4. Gas Chromatography-Mass Spectrometry Analysis of the Essential Oil

EuEO was analyzed using a THERMO DSQ II GC-MS instrument (Thermo Fisher Scientific, Austin, TX, USA), under the following conditions: DB-5 ms (30 m × 0.25 mm i.d.; 0.25 *μ*m film thickness) fused silica capillary column, with the following temperature program: 60°C, subsequently increased by 3°C/min up to 240°C; injector temperature: 250°C; carrier gas: helium (high purity), adjusted to a linear velocity of 32 cm/s (measured at 100°C). The injection type was splitless. The oil sample was diluted 1 : 100 in hexane solution, and the volume injected was 0.1 *μ*L. The split flow was adjusted to yield a 2 : 1000 ratio, and the septum sweep was constant at 10 mL/min. All mass spectra were acquired in electron impact (EI) mode with an ionization voltage of 70 eV over a mass scan range of 35–450 amu. The temperature of the ion source and connection parts was 200°C.

### 2.5. Identification and Quantification of Constituents

The quantitative data regarding the volatile constituents were obtained by peak-area normalization using a FOCUS GC/FID operated under conditions similar to those used in the GC-MS assay, except for the carrier gas, which was nitrogen. The retention index was calculated for all the volatile constituents by using an *n*-alkane (C_8_–C_20_) homologous series. When possible, individual components were identified by coinjection with authentic standards. Otherwise, the peak assignment was performed by comparison of both mass spectrum and GC retention data by using authentic compounds previously analyzed and stored in our private library, as well as with the aid of commercial libraries containing retention indices and mass spectra of volatile compounds commonly found in essential oils [23, 24]. Percentage (relative) of the identified compounds was computed from the GC peak area.

### 2.6. Parasites and Mice

*Leishmania* (*Leishmania*) *amazonensis* (IFLA/BR/67/PH8) was used for the determination of the anti-*Leishmania* activity. Parasites were grown in supplemented Schneider's medium (10% heat-inactivated fetal bovine serum (FBS), 100 U·mL^−1^ penicillin, and 100 *μ*g/mL streptomycin at 26°C). Murine macrophages were collected from the peritoneal cavities of male and female BALB/c mice (4-5 weeks old) from Medicinal Plants Research Center (NPPM/CCS/UFPI), located at Teresina, PI, Brazil. The macrophages were maintained at a controlled temperature (24 ± 1°C) and light conditions (12 h light/dark cycle). All protocols were approved by the Animal Research Ethics Committee (CEEAPI no. 001/2012). 

### 2.7. Anti-Leishmania Activity Assay

Promastigotes in the logarithmic growth phase were seeded in 96-well cell culture plates at 1 × 10^6^ *Leishmania* per well. Then, essential oil was added to the wells in serial dilutions of 400, 200, 100, 50, 25, 12.5, 6.25, and 3.12 *μ*g·mL^−1^. The plate was kept at 26°C in a biological oxygen demand (BOD) incubator, and *Leishmania* was observed and counted by using a Neubauer hemocytometer after 24, 48, and 72 h to monitor growth and viability [25]. Assays were performed in triplicate and were repeated 3 times on different days.

### 2.8. Cytotoxicity Determination

Cytotoxicity of EuEO was assessed using the MTT test. In a 96-well plate, 100 *μ*L of supplemented RPMI 1640 medium and about 1 × 10^5^ macrophages were added per well. They were then incubated at 37°C in 5% of CO_2_ for 2 h to allow cell adhesion. After this time, 2 washes with supplemented RPMI 1640 medium were performed to remove cells that did not adhere. Subsequently, EuEO was added, in triplicate, after being previously diluted in supplemented RPMI 1640 medium to a final volume of 100 *μ*L for each well at the tested concentrations (100, 50, 25, 12.5, 6.25, and 3.12 *μ*g·mL^−1^). Cells were then incubated for 48 h. At the end of the incubation, 10 *μ*L of MTT diluted in PBS was added at a final concentration of 5 mg·mL^−1^ (10% of volume, i.e., 10 *μ*L for each 100 *μ*L well) and was incubated for an additional 4 h at 37°C in 5% CO_2_. The supernatant was then discarded, and 100 *μ*L of DMSO was added to all wells. The plate was then stirred for about 30 min at room temperature to complete formazan dissolution. Finally, spectrophotometric reading was conducted at 550 nm in an ELISA plate reader [26].

### 2.9. Hemolytic Activity

The hemolytic activity was investigated by incubating 20 *μ*L of serially diluted essential oil in phosphate-buffered saline (PBS; 400, 200, 100, 50, 25, 12.5, 6.25, and 3.12 *μ*g·mL^−1^) with 80 *μ*L of a suspension of 5% red blood cells (human O^+^) for 1 h at 37°C in assay tubes. The reaction was slowed by adding 200 *μ*L of PBS, and then the suspension was centrifuged at 1000 g for 10 min. Cell lysis was then measured spectrophotometrically (540 nm). The absence of hemolysis (blank control) or total hemolysis (positive control) was determined by replacing the essential oil solution with an equal volume of PBS or Milli-Q sterile water, respectively. The results were determined by the percentage of hemolysis compared to the positive control (100% hemolysis), and the experiments were performed in triplicate [27].

### 2.10. Treatment of Infected Macrophages

Macrophages were collected in 24-well culture plates at a concentration of 2  × 10^5^ cells/500 *μ*L in RPMI 1640, containing sterile 13 mm round coverslips. They were then incubated at 37°C in 5% of CO_2_ for 2 h to allow cell adhesion. After this time, the medium was replaced with 500 *μ*L of supplemented RPMI 1640 and incubated for 4 h. The medium was then aspirated, and a new medium containing promastigotes (in the stationary phase) at a ratio of 10 promastigotes to 1 macrophage was added to each well. After 4 h of incubation in 5% CO_2_ at 37°C, the medium was aspirated to remove free promastigotes, and the test samples were added at nontoxic concentrations to the macrophages (3.12, 1.56, and 0.78 *μ*g·mL^−1^). This preparation was then incubated for 48 h, after which the coverslips were removed, fixed in methanol, and stained with Giemsa. For each coverslip, 100 cells were evaluated and both the number of infected macrophages and the amount of parasites per macrophage were counted [26].

### 2.11. Lysosomal Activity

Measurement of the lysosomal activity was carried out according to the method of Grando et al. [28]. Peritoneal macrophages were plated and incubated with EuEO in serial dilutions of 100, 50, 25, 12.5, 6.25, and 3.12 *μ*g·mL^−1^. After 4 h of incubation at 37°C in 5% CO_2_, 10 *μ*L of neutral red solution was added and incubated for 30 min. Once this time had elapsed, the supernatant was discarded, the wells were washed with 0.9% saline at 37°C, and 100 mL of extraction solution was added (glacial acetic acid 1% v/v and ethanol 50% v/v dissolved in bidistilled water) to solubilize the neutral red inside the lysosomal secretion vesicles. After 30 min on a Kline shaker (model AK 0506), the plate was read at 550 nm by using an ELISA plate reader.

### 2.12. Phagocytosis Test

Peritoneal macrophages were plated and incubated with EuEO in serial dilutions of 100, 50, 25, 12.5, 6.25, and 3.12 *μ*g·mL^−1^. After 48 h of incubation at 37°C in 5% CO_2_, 10 *μ*L of zymosan color solution was added and incubated for 30 min at 37°C. After this, 100 mL of Baker's fixative (formaldehyde 4% v/v, sodium chloride 2% w/v, and calcium acetate 1% w/v in distilled water) was added to stop the phagocytosis process. Thirty minutes later, the plate was washed with 0.9% saline in order to remove zymosan that was not phagocytosed by macrophages. The supernatant was removed and added to 100 mL of extraction solution. After solubilization in a Kline shaker, the absorbances were measured at 550 nm by using an ELISA plate reader [28].

### 2.13. Nitric Oxide (NO) Production

In 96-well plates, 2 × 10^5^ macrophages were added per well and incubated at 37°C in 5% CO_2_ for 4 h to allow cell adhesion. A new medium containing promastigotes (in the stationary phase) at a ratio of 10 promastigotes per macrophage was added to half of the wells. The essential oil was added after being previously diluted in a culture medium containing different concentrations (100, 50, 25, 12.5, 6.25, and 3.12 *μ*g·mL^−1^) in the absence or presence of *L. amazonensis*, and was then incubated again at 37°C in 5% CO_2_ for 24 h. After this period, the cell culture supernatant was collected and transferred to another plate for measurement of nitrite. A standard curve was prepared with 150 *μ*M sodium nitrite in Milli-Q water at varying concentrations of 1, 5, 10, 25, 50, 75, 100, and 150 *μ*M diluted in a culture medium. At the time of dosing, 100 *μ*L of either the samples or the solutions prepared for obtaining the standard curve was mixed with an equal volume of Griess reagent. The analysis was performed using an ELISA plate reader at 550 nm [28].

### 2.14. Statistical Analysis

All assays were performed in triplicate and in 3 independent experiments. The half-maximal inhibitory concentration (IC_50_) values and 50% cytotoxicity concentration (CC_50_) values, with 95% confidence intervals, were calculated using a probit regression model and Student's *t*-test. Analysis of variance (ANOVA) followed by a Bonferroni test were performed, taking a *P* value of <0.05 as the minimum level required for statistical significance.

## 3. Results

### 3.1. Analysis of the Essential Oil

The yield of EuEO was 0.3%. Thirty-two components were identified in this oil by GC-MS, constituting 92.65% of the total mixture. EuEO was shown to be rich in oxygenated sesquiterpenes (62.55%) and sesquiterpene hydrocarbons (29.37%). Curzerene was the major constituent (47.3%), followed by *γ*-elemene (14.25%) and *trans*-*β*-elemenone (10.4%). The chemical constituents are listed in Table 1.

### 3.2. Anti-Leishmania Activity Assay

**Figure 1:**
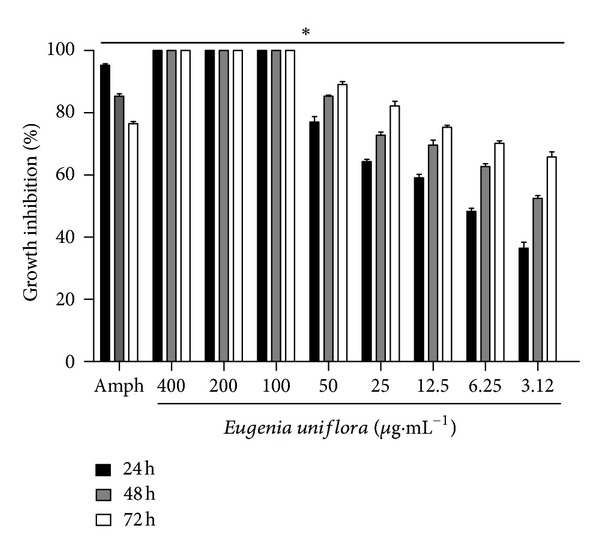
Effect of *Eugenia uniflora* essential oil (400, 200, 100, 50, 25, 12.5, 6.25, and 3.12 *μ*g·mL^−1^) or amphotericin B (Amph) (2 *μ*g·mL^−1^) on *Leishmania amazonensis* promastigotes. Cultures of log-phase promastigotes (1 × 10^6^) were incubated at 26°C for 24, 48, and 72 h in different essential oil concentrations. Data represent the mean percentage of growth inhibition ± standard error of 3 experiments carried out in triplicate. *P < 0.05.

The inhibitory profile of EuEO on *L. amazonensis* promastigotes showed a significant concentration-dependent decrease (*P* < 0.05) in parasite viability, with 100% inhibition of promastigote growth at concentrations of 400, 200, and 100 *μ*g·mL^−1^ (Figure 1). The IC_50_ was 1.75 *μ*g·mL^−1^ at 72 h of exposure (Table 2). In cultures treated with EuEO, we used an optical microscope to observe morphological changes in the promastigotes, such as cells with rounded or completely spherical shapes, as well as cellular debris, in contrast to the spindle forms present in the control (data not shown). Amphotericin B was used as a positive control and was tested at a concentration of 2 *μ*g·mL^−1^. The highest inhibitory effect of amphotericin B was observed after 24 h.

### 3.3. Cytotoxicity and Hemolysis Assay

**Figure 2:**
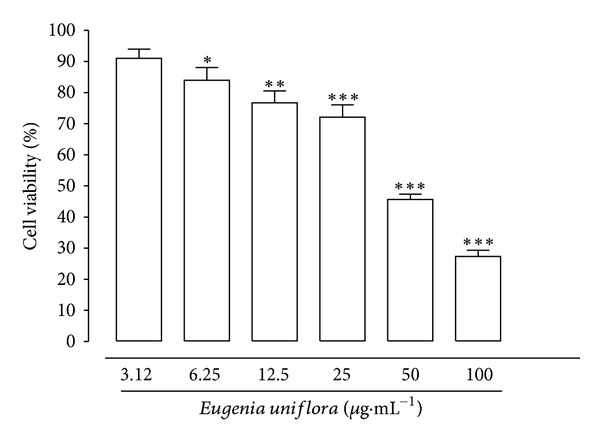
Cytotoxicity of *Eugenia uniflora* essential oil on the viability of murine peritoneal macrophages. Peritoneal macrophages were seeded at 1 × 10^5^/well in 96-well microplates and incubated for 48 h in the presence of *E. uniflora* L. essential oil at concentrations of 100, 50, 25, 12.5, 6.25, and 3.12 *μ*g·mL^−1^. Viability was determined with 3-(4,5-dimethylthiazol-2-yl)2,5-diphenyltetrazolium bromide (MTT), and the optical density was determined at 540 nm. Data represent the mean parasite density ± standard error of 3 experiments carried out in triplicate. *P < 0.05; **P < 0.01; and ***P < 0.001.

**Figure 3:**
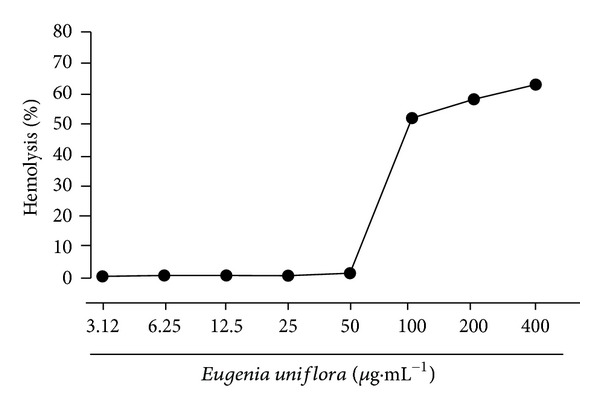
Hemolytic activity of *Eugenia uniflora* essential oil in a 4% suspension of human O^+^ red blood cells after 1 h of incubation.

The cytotoxicity of EuEO for murine peritoneal macrophages and erythrocytes is shown in Figures 2 and 3, respectively, and in Table 2. The EO from *E. uniflora* significantly altered (*P* < 0.05) macrophage viability at a concentration of 6.25 *μ*g·mL^−1^ and was able to reduce 50% of the macrophage viability (CC_50_) at 45.3 *μ*g·mL^−1^. The cytotoxicity of EO from *E. uniflora* for human blood type O^+^ erythrocytes was 63.22% at the highest concentration tested (400 *μ*g·mL^−1^); however, it appeared to be nontoxic at concentrations less than 50 *μ*g·mL^−1^ (Figure 3).

### 3.4. Treatment of Infected Macrophages

**Figure 4:**
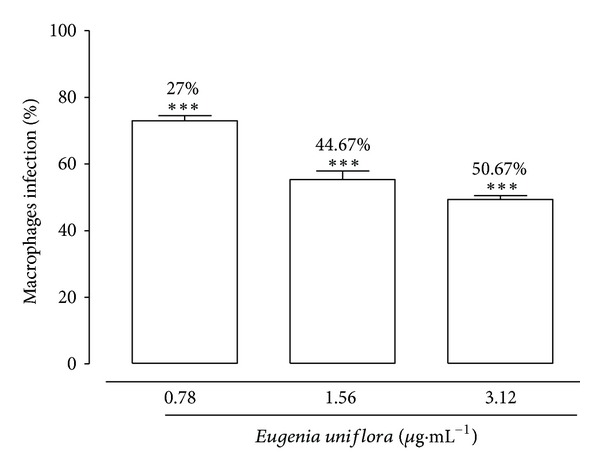
Effect of *Eugenia uniflora* essential oil on macrophage infection after 48 h of exposure. Peritoneal macrophage cells were infected with promastigotes of *Leishmania amazonensis* and then treated with 3.12, 1.56, or 0.78 *μ*g·mL^−1^ of *Eugenia uniflora* essential oil. Data represent the mean parasite density ± standard error of 3 experiments carried out in triplicate. ***P < 0.001.

**Figure 5:**
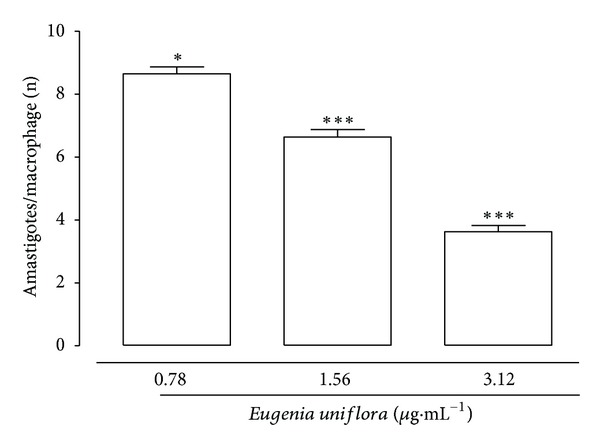
Effect of *Eugenia uniflora* essential oil on the survival of *Leishmania amazonensis* amastigotes internalized in macrophages. *Leishmania amazonensis*-infected mouse peritoneal macrophages were treated with 3.12, 1.56, or 0.78 *μ*g·mL^−1^ of *Eugenia uniflora* essential oil. After 48 h of incubation, amastigote survival was assessed. Data represent the mean parasite density ± standard error of 3 experiments carried out in triplicate. ***P < 0.001.

Figures 4 and 5 show the results of EuEO treatment of macrophages infected with *L. amazonensis*. The values obtained revealed a significant and concentration-dependent reduction in macrophage infection at 48 h with all the 3 EuEO concentrations studied. The reduction of infection was 27%, 44.67%, and 50.67% at 0.78, 1.56, and 3.12 *μ*g·mL^−1^, respectively. EuEO also decreased the number of amastigotes per infected cell in a concentration-dependent manner (Figure 5). The IC_50_ at 48 h of exposure to EuEO was 1.92 *μ*g·mL^−1^ (Table 2). These values and the CC_50_ values for macrophages were used to calculate the selectivity index that is useful for assessing the safety level of EuEO in mammalian cells (Table 2). This index represents the relationship between CC_50_ and IC_50_ for amastigotes.

### 3.5. Lysosomal Activity and Phagocytosis Assay

**Figure 6:**
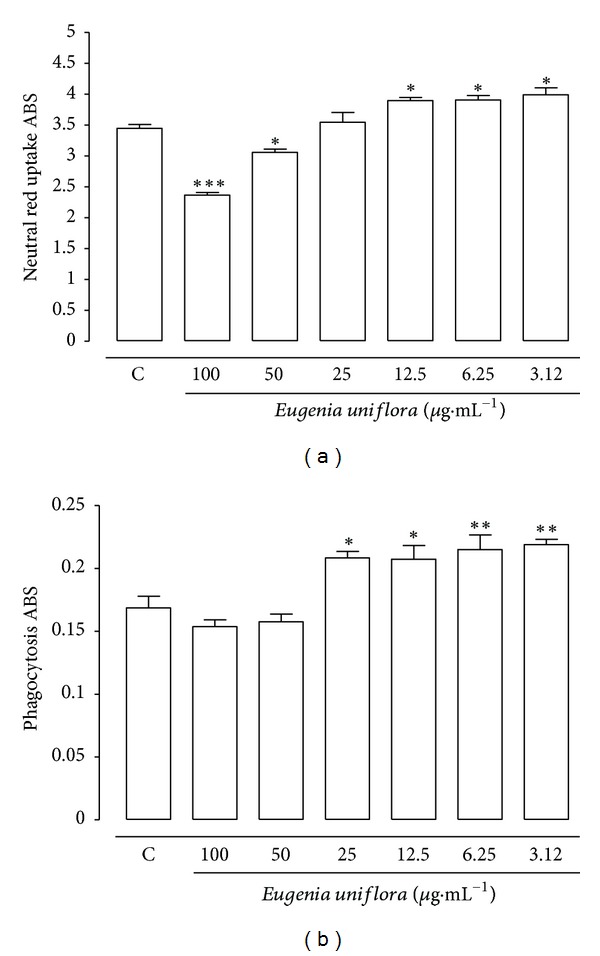
The influence of *Eugenia uniflora* essential oil on the lysosomal activity (a) and phagocytic activity (b) of peritoneal macrophages at concentrations of 100, 50, 25, 12.5, 6.25, and 3.12 *μ*g·mL^−1^. Data represent the mean density ± standard error of 3 experiments carried out in triplicate. *P < 0.05 and ***P < 0.001. C: control. ABS: absorbance.

Lysosomal activity was assessed based on the retention of neutral red in macrophage lysosomes and was determined colorimetrically. The lysosomal activity of macrophages treated with EuEO showed significant reduction at concentrations of 100 and 50 *μ*g·mL^−1^ (Figure 6(a)) due to cytotoxicity (CC_50_ 45.3 *μ*g·mL^−1^). However, we observed a statistically significant increase in lysosomal activity at concentrations from 12.5 to 3.12 *μ*g·mL^−1^ compared to control. Another parameter reflecting macrophage activation was the phagocytosis of stained zymosan when the cells were exposed to an external stimulus. The phagocytic ability of macrophages incubated with EuEO is shown in Figure 6(b). There was a significant increase in phagocytosis of zymosan particles in the wells treated with EuEO at concentrations ranging from 25 to 3.12 *μ*g·mL^−1^.

### 3.6. NO Production

**Figure 7:**
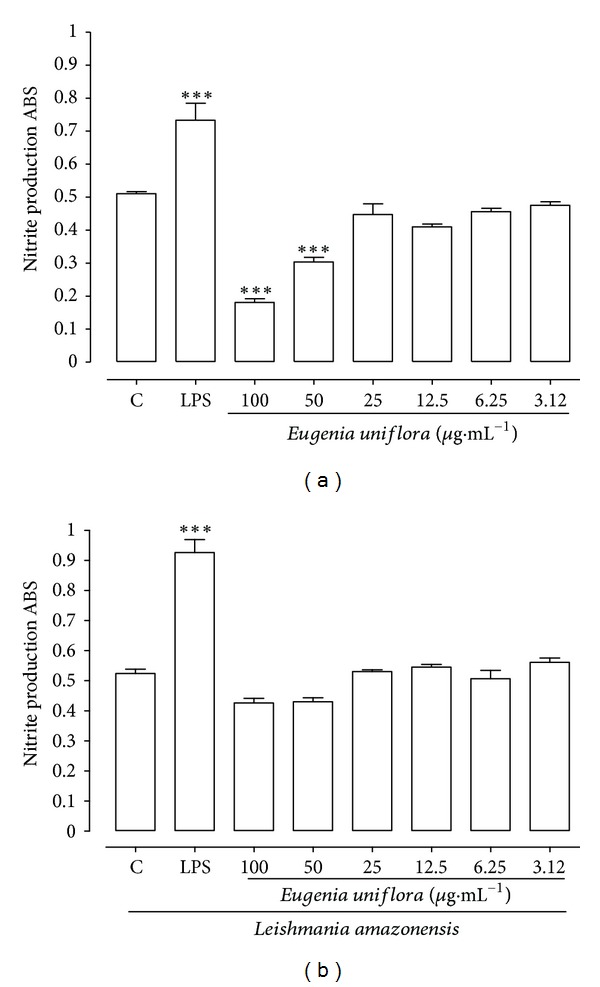
Production of nitric oxide (NO). Murine macrophages (2 × 10^5^) were treated with *Eugenia uniflora* essential oil (100, 50, 25, 12.5, 6.25, and 3.12 *μ*g·mL^−1^) in the absence (a) or presence of *Leishmania amazonensis* (b) over 24 h. Data represent the mean density ± standard error of 3 experiments carried out in triplicate. ***P < 0.001. C: control. ABS: absorbance.

NO production was determined indirectly by measuring nitrite produced by macrophages treated with EuEO and stimulated (or not stimulated) by *L. amazonensis* promastigotes. Macrophages treated with EuEO, but not stimulated by *Leishmania*, showed significant reduction in NO production at concentrations of 100 and 50 *μ*g·mL^−1^ (Figure 7(a)), while macrophages stimulated by the parasite showed increased NO at a concentration of 3.12 *μ*g·mL^−1^, albeit without statistical significance (*P* > 0.05) (Figure 7(b)).

## 4. Discussion

The need to identify new compounds with anti-*Leishmania* properties that are more effective and less toxic than conventional drugs has motivated research on natural products isolated from plant species. These substances predominantly consist of alkaloids, terpenes, flavonoids, benzopyrans, phenolics, and sesquiterpene lactones and have been identified in plant species with documented anti-*Leishmania* activity [29, 30]. The chemical composition of EuEO obtained in this study revealed sesquiterpenes to be the dominant chemical class, corroborating the findings of many previous studies [16, 31–33]. Despite the prevalence of sesquiterpenes, the species has different chemotypes, with different major constituents being observed in different studies. The specimen analyzed in this study had curzerene as the major constituent, which again corroborated previous reported findings [32, 34]. According to Adio [35], germacrone is a heat-sensitive compound that may degrade to *trans*-*β*-elemenone during GC analysis. This may be the explanation of the presence of *trans*-*β*-elemenone among the major compounds of EuEO. Some germacrone products have shown pharmacological activity, as antitumor [35].

 According to Costa et al. [20] the composition of the essential oil from *E. uniflora* leaves might be influenced by fruit colors. The plants with red fruit colors have curzerene as the major component as in our studied plant. In Maranhão, where the samples were collected, the majority of plants of *E. uniflora* have red fruit color [32]. Selina-1,3,7(11)-trien-8-one [36], germacrene B [16, 33], and *α*- and *β*-selinene [37] have been found to be the major constituents of other *E. uniflora* chemotypes. Genetic variability, geographical and environmental conditions, and the method of environmental management directly influence the yield and composition of essential oils [38]. It is the first report of essential oil analyzed from *E. uniflora* collected in São Luís, MA, Brazil and this could be an excellent source of natural product with interest for pharmaceutical industry.

The present study was conducted to evaluate the anti-*Leishmania* activity of EuEO on promastigotes and amastigotes of *L. amazonensis*, its potential mechanisms of macrophage activation, and its cytotoxicity in mammalian cells. The EuEO showed significant concentration-dependent activity against promastigotes of *L. amazonensis*, with an IC_50_ of 1.75 *μ*g·mL^−1^. Previous studies have suggested that these classes of natural products may have potential anti-*Leishmania* activity. *β*-Caryophyllene (a sesquiterpene hydrocarbon) and nerolidol (an oxygenated sesquiterpene) are examples of terpenic substances with well-characterized anti-*Leishmania* activity, possibly associated with inhibition of cellular isoprenoid biosynthesis [8, 39].

It has been shown that the lipophilic components of essential oils may affect layers of polysaccharides, fatty acids, and phospholipids in plasma membranes of promastigotes of *Leishmania* spp. This then leads to cell lysis and release of macromolecules [40]. In the cytoplasm, these substances can disrupt the specific metabolic pathways of lipids and proteins or stimulate depolarization of mitochondrial membranes, which can lead to cell necrosis or apoptosis [41, 42].

Amphotericin B is a drug of choice for the treatment of different forms of leishmaniasis. Because of this it was used as a positive control. We observed a significant inhibitory effect on promastigote growth after 24 h of exposure to amphotericin B, but leading to an increase in viable promastigote forms at 48 and 72 h. Similar results were observed in previous studies [43]. Some *Leishmania* strains have shown mechanisms of resistance to amphotericin B. This may has happened with the strain used in this study. The reproduction of resistant parasites may have led to a higher cell viability after 48 and 72 h [44, 45].

Experimental models using amastigotes internalized into macrophages can produce results that more closely approximate those obtained using animal models, since these forms are responsible for the clinical manifestations of leishmaniasis [46]. Amastigotes can be found in parasitophorous vacuoles of infected macrophages; therefore, a therapeutic drug will only be effective against *Leishmania* spp. if it can cross the host cell membrane and act on the amastigotes inside the vacuole. Thus, assays of activity against intracellular amastigotes are the most effective way to relate the *in vitro* activity of a possible substance with its *in vivo* effectiveness [47]. The inhibition of amastigote growth was even greater at 48 h after exposure to oil, with an IC_50_ of 1.92 *μ*g/mL, whereas the IC_50_ for promastigotes was 3.04 *μ*g·mL^−1^ for the same duration of exposure. Previous studies demonstrated greater activity against amastigotes versus promastigotes of essential oils of *Artemisia absinthium* and *Satureja punctata* [48].

Mechanisms of anti-*Leishmania* action on *Leishmania* spp. amastigotes have also been described for essential oils and their terpene compounds. Monoterpenes such as linalool, isolated from leaves of *Croton cajucara* (Euphorbiaceae), significantly increased NO production in macrophages infected with *L. amazonensis* and acts directly on the parasite, as evidenced by mitochondrial swelling and changes in the kinetoplast and the organization of nuclear chromatin [6]. In this study, the production of NO was determined by measuring nitrite. The results from macrophages treated with EuEO revealed that EuEO does not induce NO production, which demonstrates that its promising anti-*Leishmania* activity does not occur by this route.

Natural products that are able to activate macrophages, and are thus biological response modifiers, have been extensively studied [49, 50]. In this context, the EuEO demonstrated potential in the activation of macrophages by increasing phagocytic capacity and lysosomal activity, immunomodulatory activities possibly involved in its anti-*Leishmania* activity. Phagocytosis and the lysosomal system are critical to macrophage function, because of their roles in internalization, degradation, and eventually presentation of peptides derived from antigens required for host defense. In addition, antigen presentation occurs through phagocytosis and endosomal/lysosomal targeting systems [51–53]. 

Due to the need for anti-*Leishmania* substances that are more selective for the parasite and less toxic to host cells and since EuEO showed low IC_50_ values, it was important to investigate the cytotoxic activity of EuEO against mammalian cells. EuEO showed significant toxicity in macrophages, with a CC_50_ of 45.3 *μ*g·mL^−1^, but when compared with its IC_50_ value in amastigotes, this indicates a secure selectivity index above 20. The literature recommends that for amastigotes internalized in macrophages, this index must be demonstrated as near to or greater than 20 [54]. The investigation of the toxic action of new substances with anti-*Leishmania* activity on macrophages is important, since they are the main cells of the vertebrate host parasitized by *Leishmania* spp. [55, 56]. Furthermore, various other cytotoxic assays can be conducted using animal and human cells to determine the safety of a test substance, leading to a future treatment *in vivo*. Tests performed with erythrocytes enable assessment of a drug's potential to cause injury to the plasma membrane of a cell, either by forming pores or by total rupture, leading to cellular damage or changes in membrane permeability [57]. This is a model used for the preliminary study of the protective and toxic effects of substances, being a possible indicator of this type of damage to cells *in vivo* [58–60]. The results obtained in this test with EuEO revealed low toxicity to erythrocytes, since EuEO showed 0% hemolysis at concentrations of 50 *μ*g·mL^−1^ and lower.

## 5. Conclusions

In conclusion, the data in this study show that the essential oil of leaves from *E. uniflora* (EuEO), characterized by sesquiterpenes, has anti-*Leishmania* activity in both stages of *L. amazonensis*. This study also demonstrated that EuEO activity is not mediated by NO production. On the other hand, activation of macrophages may be involved in the mechanisms of EuEO anti-*Leishmania* activity, as evidenced by increases in both phagocytic capacity and the lysosomal compartment. Further investigation is needed to determine additional mechanisms involved in the anti-*Leishmania* activity of this plant species, as well as *in vivo* studies in models of leishmaniasis.

## Figures and Tables

**Table 1:** Chemical composition and retention indices of the constituents of *Eugenia uniflora* essential oil.

Constituents^a^	RRI		% A^d^
	Cal.^b^	Lit.^c^
Myrcene	989	988	0.19
Limonene	1026	1029	0.18
Linalool	1095	1095	0.16
(*Z*)-3-Hexenyl butyrate	1186	1184	0.10
Cuminaldehyde	1240	1238	0.10
*δ*-Elemene	1335	1338	1.32
*α*-Cubebene	1349	1345	0.10
*β*-Elemene	1390	1389	5.51
Sativene	1394	1390	0.06
(*E*)-Caryophyllene	1417	1417	4.33
*trans*-*α*-Bergamotene	1437	1432	0.05
***γ*-Elemene**	**1434**	**1434**	**14.25**
Aromadendrene	1440	1439	0.07
*α*-Humulene	1455	1452	0.21
Alloaromadendrene	1462	1458	0.11
*β*-Chamigrene	1475	1476	0.38
Germacrene D	1482	1484	1.19
*β*-Selinene	1487	1489	0.70
**Curzerene**	**1498**	**1499**	**47.3**
*γ*-Cadinene	1513	1513	0.12
*δ*-Cadinene	1523	1522	0.28
Selina-3,7-(11)-diene	1546	1545	0.24
Germacrene B	1558	1559	0.45
Spathulenol	1578	1577	0.17
Caryophyllene oxide	1581	1582	0.13
Globulol	1585	1590	0.25
Viridiflorol	1590	1592	0.08
***trans*-*β*-Elemenone**	**1597**	**1601**	**10.4**
Atractilona	1655	1657	2.38
Germacrone	1694	1693	1.51
Eudesm-7(11)-en-4-ol	1700	1700	0.33

Monoterpene hydrocarbons	0.37		
Oxygenated monoterpenes	0.36		
Sesquiterpene hydrocarbons	29.37		
Oxygenated sesquiterpenes	62.55		

Total	92.65		

^a^Compounds listed in order of elution on the DB-5ms column. ^b^Relative retention indices (RRIs) experimentally determined against *n*-alkanes by using the DB-5ms column. ^c^RRIs reported in the literature [23, 24]. ^d^Content expressed as percentages obtained by integration of the GC peak area.

**Table 2:** Anti-*Leishmania* and cytotoxic effects of *Eugenia uniflora* essential oil.

	Macrophage	Promastigote				Amastigote	
	CC_50_ (**μ**g·mL^−1^)	IC_50 _(**μ**g·mL^−1^)			SI	IC_50_ (**μ**g·mL^−1^)	SI
	48 h	24 h	48 h	72 h		48 h
EuEO	45.3 ± 2.45	6.96 ± 1.02	3.04 ± 0.75	1.75 ± 0.53	14.9^a^	1.92 ± 0.8	23.59^b^

^a^SI_pro_ (selectivity index) = CC_50_ macrophages/IC_50_ promastigote forms (48 h).

^b^SI_ama_ (selectivity index) = CC_50_ macrophages/IC_50_ amastigote forms.
